# London Prices of Drugs.—May 27

**Published:** 1814-06

**Authors:** 


### LONDON PRICES OF DRUGS.—May 27.

*(To be continued regularly.)*

| | £ | s | d. | £. | s. | d. | per |
|---|---|---|---|---|---|---|---|
| Aloes Barbadoes, from | 20 | 10 | 0 to | 21 | 0 | 0 | C. |
| —— Cape | 10 | 0 | 0 | 11 | 0 | 0 | — |
| —— Succotrina | 20 | 0 | 0 | 0 | 0 | 0 | — |
| —— Epatica or E.I. | 14 | 0 | 0 | 16 | 0 | 0 | — |
| Angelica Root | 10 | 10 | 0 | 0 | 0 | 0 | — |
| Alkanet Root | 6 | 0 | 0 | 0 | 0 | 0 | — |
| Antimony Crude | 2 | 16 | 0 | 0 | 0 | 0 | — |
| Aqua Fortis | S. | 0 | 8 | D. | 1 | 1 | lb |
| Arrow Root fine | 0 | 2 | 0 | 0 | 3 | 6 | — |
| —— ordinary | 0 | 1 | 0 | 0 | 1 | 6 | — |
| Arsenic Red | 5 | 0 | 0 | 0 | 0 | 0 | C. |
| —— White | 3 | 0 | 0 | 0 | 0 | 0 | — |
| Balsam Capivi | 0 | 6 | 0 | 0 | 6 | 3 | lb. |
| —— Peru | 1 | 0 | 0 | 1 | 1 | 0 | — |
| —— Tolu | 0 | 14 | 0 | 0 | 14 | 6 | — |
| Bark Jesuits', Com. | 0 | 3 | 6 | 0 | 4 | 0 | — |
| —— Second | 0 | 6 | 0 | 0 | 7 | 0 | — |
| —— Quil or best | 0 | 8 | 0 | 0 | 10 | 0 | — |
| —— Red | 0 | 7 | 0 | 0 | 10 | 0 | — |
| —— Yellow | 0 | 4 | 6 | 0 | 5 | 0 | — |
| Borax refined E.I. | none. | | | | | | |
| —— English | 0 | 3 | 9 | 0 | 4 | 0 | lb. |
| —— unrefined or Tinc. | 10 | 0 | 0 | 11 | 0 | 0 | C. |
| Camphire refined | 0 | 8 | 9 | 0 | 9 | 0 | lb. |
| —— unrefined | 28 | 0 | 0 | 32 | 0 | 0 | C. |
| Cantharides | 8 | 9 | 0 | 0 | 0 | 0 | lb. |
| Cardamoms (best) | 0 | 7 | 0 | 0 | 12 | 0 | — |
| Cassia Buds | 36 | 0 | 0 | in bond | 0 | 0 | C. |
| —— Fistula W.I. | 6 | 10 | 0 | in bond | | | — |
| —— Lignea | 25 | 0 | 0 | in bond | 0 | 0 | — |
| Castoreum American | 2 | 10 | 0 | 2 | 15 | 0 | lb. |
| —— Russia | 11 | 0 | 0 | 0 | 0 | 0 | — |
| Castor Oil per bottle 1½ lb. | 0 | 3 | 9 | 0 | 4 | 3 | bo |
| Cocculus Indicus | 6 | 0 | 0 | 7 | 0 | 0 | C. |
| Colocynth Turkey | 0 | 5 | 0 | 0 | 5 | 6 | lb. |
| Columbo Root | 4 | 15 | 0 | 6 | 0 | 0 | C. |
| Cream of Tartar | 6 | 10 | 0 | 7 | 0 | 0 | — |
| Gallangal East-India | 5 | 0 | 0 | 0 | 0 | 0 | C. |
| Gentian Root | 3 | 5 | 0 | 3 | 6 | 0 | — |
| Ginseng | 0 | 1 | 6 | 0 | 0 | 0 | lb. |
| Grains of Guinea | 6 | 6 | 0 | 6 | 10 | 0 | C. |
| Gum Ammo. Drop | 30 | 0 | 0 | 0 | 0 | 0 | — |
| —— Lump | 8 | 0 | 0 | 9 | 0 | 0 | — |
| —— Animi | 3 | 10 | 0 | 7 | 10 | 0 | — |
| —— Arabic E.I. | 2 | 0 | 0 | 4 | 0 | 0 | — |
| —— Turkey Fine | 10 | 10 | 0 | 14 | 14 | 0 | — |
| —— Barbary | 7 | 7 | 0 | 8 | 0 | 0 | — |
| —— Assafœtida | 10 | 0 | 0 | 15 | 0 | 0 | — |
| —— Benjamin | 8 | 10 | 0 | 35 | 0 | 0 | — |
| —— Cambogium | 20 | 0 | 0 | 26 | 0 | 0 | — |
| —— Copal scraped | 0 | 3 | 0 | 0 | 4 | 6 | lb. |
| —— Galbanum | 16 | 0 | 0 | 20 | 0 | 0 | C. |

| | £. | s. | d. | £. | s. | d. | per |
|---|---|---|---|---|---|---|---|
| Gum Guaiacum, from | 0 | 2 | 6 to | 0 | 3 | 0 | lb. |
| —— Mastic | 0 | 4 | 6 | 0 | 4 | 9 | — |
| —— Myrrh | 15 | 0 | 0 | 18 | 0 | 0 | C. |
| —— Olibanum | 6 | 0 | 0 | 7 | 0 | 0 | — |
| —— Oppoponax | 80 | 0 | 0 | 0 | 0 | 0 | — |
| —— Sandrac | 9 | 0 | 0 | 10 | 0 | 0 | — |
| —— Seneca Garbled | 5 | 2 | 0 | 5 | 5 | 0 | — |
| —— Tragacanth | 36 | 0 | 0 | 38 | 0 | 0 | — |
| Jalap | 0 | 5 | 0 | 0 | 5 | 3 | lb. |
| Ipecacuanha | 1 | 0 | 0 | 1 | 1 | 0 | — |
| Isinglass Book | 0 | 10 | 6 | 0 | 11 | 0 | — |
| —— Leaf | 0 | 10 | 6 | 0 | 11 | 0 | — |
| —— Long Staple | 0 | 10 | 6 | 0 | 11 | 0 | — |
| —— Short Staple | 0 | 10 | 6 | 0 | 11 | 0 | — |
| Manna Flakey | 0 | 5 | 3 | 0 | 5 | 6 | — |
| —— Sicily in Sorts | 0 | 4 | 0 | 0 | 4 | 3 | — |
| Musk China | 0 | 18 | 0 | 1 | 0 | 0 | oz. |
| Nux Vomica | 3 | 0 | 0 | 3 | 10 | 0 | C. |
| Oil of Vitriol | 0 | 0 | 3¾ | 0 | 0 | 0 | lb. |
| Opium East-India | 1 | 2 | 0 | 1 | 3 | 0 | — |
| —— Turkey | 1 | 9 | 0 | 1 | 10 | 0 | — |
| Pink Root | 0 | 4 | 0 | 0 | 4 | 6 | — |
| Quicksilver | 0 | 4 | 10 | 0 | 4 | 11 | — |
| Rhubarb East-India | 0 | 6 | 0 | 0 | 10 | 0 | — |
| —— Russia | 0 | 18 | 0 | 1 | 0 | 0 | — |
| Saffron Spanish | 2 | 5 | 0 | 2 | 10 | 0 | — |
| —— French | 1 | 15 | 0 | 2 | 0 | 0 | — |
| Sago | 7 | 0 | 0 | 8 | 0 | 0 | C. |
| Sal. Ammoniac | 10 | 10 | 0 | 11 | 0 | 0 | — |
| Sarsaparilla | 0 | 3 | 0 | 0 | 3 | 6 | lb. |
| Sassafras | 7 | 0 | 0 | 8 | 0 | 0 | T. |
| Scammony Aleppo | 1 | 6 | 0 | 1 | 7 | 0 | lb. |
| —— Smyrna | 0 | 10 | 0 | 0 | 12 | 0 | — |
| Senna | 0 | 3 | 0 | 0 | 3 | 6 | — |
| Seeds Anni Alicant | 6 | 0 | 0 | 6 | 10 | 0 | C. |
| —— Coriander English | 1 | 10 | 0 | 1 | 16 | 0 | — |
| —— Cummin | 7 | 0 | 0 | 7 | 10 | 0 | — |
| —— Fenugreek | 1 | 10 | 0 | 1 | 13 | 0 | — |
| Shellack | 7 | 10 | 0 | 10 | 0 | 0 | — |
| Sticklack | 6 | 0 | 0 | 8 | 0 | 0 | — |
| Snake Root | 0 | 0 | 0 | 0 | 0 | 0 | lb. |
| Soap Castile or Spanish | 12 | 0 | 0 | 14 | 0 | 0 | C. |
| Spermaceti refined | 0 | 2 | 3 | 0 | 0 | 0 | lb. |
| Tamarinds West-India | 8 | 10 | 0 | 9 | 0 | 0 | C. |
| Tapioca Lisbon | 0 | 0 | 8 | 0 | 1 | 0 | lb. |
| Turmeric Bengal | 5 | 10 | 0 | 0 | 0 | 0 | C. |
| —— China | 6 | 10 | 0 | 7 | 0 | 0 | — |
| —— West-India | 8 | 0 | 0 | 9 | 0 | 0 | — |
| Verdigris Wet | 0 | 3 | 6 | 0 | 0 | 0 | lb. |
| —— Dry | 0 | 4 | 9 | 0 | 5 | 0 | — |
| —— Crystallized | 0 | 8 | 0 | 0 | 0 | 0 | — |
| Vitriol Roman | 0 | 0 | 7 | 0 | 0 | 0 | — |
| —— Foreign white | 3 | 15 | 0 | 4 | 0 | 0 | C. |

*Price of Vials per Gross.*—8 oz. 70s.—6 oz. 58s.—4 oz. 47s.—3 oz. 43s.—2 oz. 36s.—1 oz. 30s.—½ oz. 24s.

### OBSERVATIONS.

At recent Sales of Merchandize by Auction, Messrs. Bowden and Tucker sold, on the 26th of May, 56 chests Pale Peruvian Bark, bonded, 1s. 2d. to 4s. 9d. per lb.—13 chests Red Bark, bonded, 4s. to 5s. 7d. per lb.—20 chests Pale Bark, 1s. 3d. to 3s. per lb.—3 cases Turkey Myrrh, 23l. per cwt.—28 chests Camphire, 18l. 15s. to 20l. per cwt.—7 barrels Balsam Capivi, 3s. 10d. to 4s. 5d. per lb.—6 chests Bark, 3s. to 6s. 9d. per lb.—15 cases and 12 barrels Isinglass, 7s. 10d. to 10s. 2d. per lb.—50 serons Barbary Gum Arabic, 95s. per cwt.—10 casks Venetian Cream of Tartar, 7l. 4s. to 8l. 2s per cwt.—4 vats Pill Boxes, 7s. per gross.—1 cask Assafœtida, 20s. per cwt.

